# The spatiotemporal distribution and prognostic factors of Japanese encephalitis in Shanxi Province, China, 2005–2022

**DOI:** 10.3389/fcimb.2023.1291816

**Published:** 2023-12-21

**Authors:** Peiyu Zheng, Zhiying Wen, Yuan Liu, Qinying Wang

**Affiliations:** ^1^ Department of Infectious Diseases, The First Hospital of Shanxi Medical University, Taiyuan, China; ^2^ Graduate School, Shanxi Medical University, Taiyuan, China; ^3^ Department of Infectious Disease Prevention and Control, Shanxi Provincial Center for Disease Control and Prevention, Taiyuan, China

**Keywords:** Japanese encephalitis, spatiotemporal, prognostic, epidemiological, nomogram

## Abstract

Japanese encephalitis (JE) is a naturally occurring localized disease caused by the Japanese encephalitis virus, which is spread by the Culex tritaeniorhynchus. China has a high rate of JE. Shanxi, located in North China, has a high prevalence of adult JE. Adult JE has more severe complications, mortality, and a higher disease burden, making it a public health issue. This retrospective study examined the dynamic epidemic changes, high-risk areas of JE, and clinical characteristics and prognostic factors of adult JE in Shanxi Province. The findings revealed that July to September was the primary epidemic season of JE and that JE cases were mainly in individuals over the age of 40. The incidence of JE from 2005 to 2022 demonstrated a positive spatial correlation with significant clustering characteristics, with high-incidence clusters in the south and southeast. Multivariate logistic regression analysis revealed that higher cerebrospinal fluid pressure, higher white blood cell counts, higher neutrophil percentage, deep coma, and lower albumin were independent factors for poor prognosis of adult JE. The developed risk prediction model holds great promise in early prognosis assessment of patients, providing a basis for clinical decision-making and early clinical intervention.

## 1 Introduction

Japanese encephalitis (JE) is a natural localized disease caused by the Japanese encephalitis virus (JEV), spread by the Culex tritaeniorhynchus, with pigs as the primary source of infection (Oliveira et al., 2018). Less than 1% of JEV-infected patients develop neurological disease, with encephalitis fatality rates ranging from 20-30%, and 30-50% of survivors continuing to have neurologic, cognitive, or psychiatric symptoms (Symptoms & Treatment | Japanese Encephalitis | CDC, 2022). JE is more prevalent in Southeast Asia and the Western Pacific. JEV transmission is seasonal throughout temperate Asia, peaking in the summer and autumn. Transmission can occur all year in the subtropics and tropics, with a surge during the rainy season. JE primarily affects children, but adult JE cases have increased due to a decrease in pediatric patients through childhood immunization programs in endemic areas, an increase in tourism to JE risk areas, and a gradual decrease in JEV neutralizing antibodies with age (Hills et al., 2023). Statistics from several countries and regions show that the age distribution of JE is steadily becoming more adult, with more severe complications and mortality (Yin et al., 2015) and a higher disease burden (Wang et al., 2021), making adult JE a public health concern.

There is no specific antiviral therapy for JE; supportive care is the primary approach to JE management. Several treatments, including dexamethasone (anti-inflammatory), IFN-α2a (antiviral), immunoglobulin (viral neutralization and anti-inflammatory), and minocycline (anti-inflammatory), have entered randomized clinical trials. However, none of these treatments improved the prognosis of JE patients (Ashraf et al., 2021). Immunoprophylaxis is considered to be the most effective approach to JE prevention. JE vaccine is derived from genotype III (GIII) of JE (Rajaiah and Kumar, 2022), with weak protection against GI and GV strains (Cao et al., 2016; Hegde and Gore, 2017), although GI, GIII, GV co-exist in China (Li et al., 2014). The GV strain is more pathogenic in mice than the GI/III (de Wispelaere et al., 2015). The outbreak of JE in Australia in 2022 suggests that novel genotypes might be introduced by migrating birds or mosquitoes and spread throughout the globe (Pham et al., 2022). Therefore, managing and preventing adult JE in endemic areas must include cost-effectiveness and practicality.

China has a high incidence of JE. Following the incorporation of the JE vaccine into the Expanded Program on Immunization, the incidence of JE decreased in most parts of China (excluding North China) (Shi et al., 2022b), while less developed economy regions have gradually become JE epidemic areas (Shi et al., 2022a). Adult JE was more prevalent in some provinces than the national average, notably among patients over 40 years old. The 6 provinces north of the Yangtze River were hotspots for adult JE, while Shanxi, in North China, had a high incidence of adult JE (Li et al., 2016). A study of adult JE in southern Shanxi Province revealed that having pigsties near dwellings is a potential risk factor for the prevalence of adult JE (Ren et al., 2017). However, there is no current description of JE distribution characteristics in Shanxi Province.

This retrospective study used geographic information system (GIS) spatial analysis and JE surveillance data between 2005 and 2022 in Shanxi Province to describe the dynamic epidemic changes and high-risk areas of JE in Shanxi Province and to manage and prevent the occurrence of JE in high-risk areas at an early stage. Furthermore, it is imperative to understand the clinical characteristics and prognostic factors of adult JE, given that early symptoms of adult JE are nonspecific and the prognosis is poor. The findings can guide the diagnosis and treatment of adult JE and conduct early intervention and prognostic estimation.

## 2 Methods

### 2.1 Study region

Shanxi Province is located in the east wing of the Loess Plateau in western North China, between 34°34’ to 40°44’ north latitude and 110°14’ to 114°33’ east longitude. It is a parallelogram oblique from northeast to southwest, having a total area of 156,700 square kilometers. The precipitation from June to August accounts for nearly 60% of the annual precipitation. It has jurisdiction over 117 counties or districts of 11 prefecture-level cities.

### 2.2 Study population and data collection

Data on JE cases from January 1, 2005 to December 31, 2022 were derived from the China Information System for Disease Control and Prevention, including age, date of onset, and clinical outcomes. Shanxi Center for Disease Control and Prevention provided the geographical information for each case.

This study retrospectively recruited adult patients diagnosed with JE from the First Hospital of Shanxi Medical University between January 2012 and December 2022 and the Yuncheng Second Hospital in 2006. All patients fulfilled the following criteria by the World Health Organization recommend: presence of clinical criteria of acute encephalitis syndrome and (1) detectable JE specific IgM in CSF or serum, or (2) evidence of seroconversion or a 4-fold increase of IgM or IgG in the convalescence phase by the ELISA method, or (3) isolation of virus from blood, CSF fluid or tissue, or (4) detection of JE-virus genome in serum, plasma, blood, CSF or tissue(Solomon et al., 2008). Patients aged <18 years, with other causes of consciousness disruption, encephalitis and meningitis, and with a significant lack of clinical data were excluded. General information (age, sex, underlying diseases), clinical characteristics (fever, headache, vomiting, convulsion, consciousness disturbance, and neurological signs), serological indicators (blood routine, liver and kidney function, ions), cerebrospinal fluid (CSF) (pressure, routine, biochemical, cytology), and brain MRI results were collected. These patients were classified into two groups according to GCS score at discharge: good outcomes (≥8 scores) and poor outcomes (<8 scores). Furthermore, the influencing factors of poor outcomes were investigated.

This retrospective study involved no personal information such as names and required no ethical statement.

### 2.3 Spatiotemporal analysis

The epidemiological characteristics of JE distribution such as annual cases and death, incidence and mortality, Monthly cumulative JE cases, and age group were plotted using Microsoft Excel 2021. The annual distribution of JE cases by county within Microsoft Excel were linked through the unique value of county level and included in the attribute table of Shanxi Province base map using ArcGIS. JE cases were classified into 6 levels: 0, 1-2, 3-5, 6-10, 11-20, and > 20 cases, with data from ArcGIS software shown on a county-level map of Shanxi Province. The yearly geographical distribution of JE was made according to the JE frequency. Add up the annual distribution of JE cases by county from January 1, 2005 to December 31, 2022 to draw spatiotemporal distribution of total JE cases. The Local Indicators of Spatial Association (LISA) was utilized to examine the spatial autocorrelation of the JE distribution and the Z-score, P-value and local Moran’s I coefficient were calculated. A local Moran’s I coefficient >0 indicates that the data have a positive spatial correlation. In contrast, a local Moran’s I coefficient <0 indicates that the data have a negative spatial correlation, and a local Moran’s I coefficient = 0 indicates that the data are random.

### 2.4 Statistical analysis

Data were analyzed using the IBM SPSS Statistics 26.0 and R4.3.1 software. Count data were described by the number of cases (percentage) and compared across groups using Chi-square tests or Fisher exact probability. Measurement data with a normal distribution were presented as mean ± standard deviation; an independent sample t-test was employed to compare the two groups. The measurement data with non-normal distribution were presented as median and interquartile distance; the Mann-Whitney U test was used to compare the two groups. The influencing factors of prognosis were examined using Logistic stepwise regression (backward elimination) to develop a prediction model. The p-values > 0.05 used for excluding variables at each step. The results were presented as odds ratio (OR) and 95% confidence interval (CI), and the forest map was generated using the “forestplot” package in R software. The predictive capacity of the prediction model was evaluated using the Receiver operating characteristic (ROC) curve, defined by area under the curve (AUC) and 95%CI. p < 0.05 (double-tailed) denoted statistical significance. The “ResourceSelection” package in R software was used to evaluate the calibration ability of the model using the calibration curve and Hosmer-Lemeshow test. The p > 0.05 indicated a good fit of the model in the Hosmer-Lemeshow test. A nomogram diagram was generated using the “rms” package.

## 3 Results

### 3.1 Epidemiological features of JE in Shanxi Province

**Figure 1:**
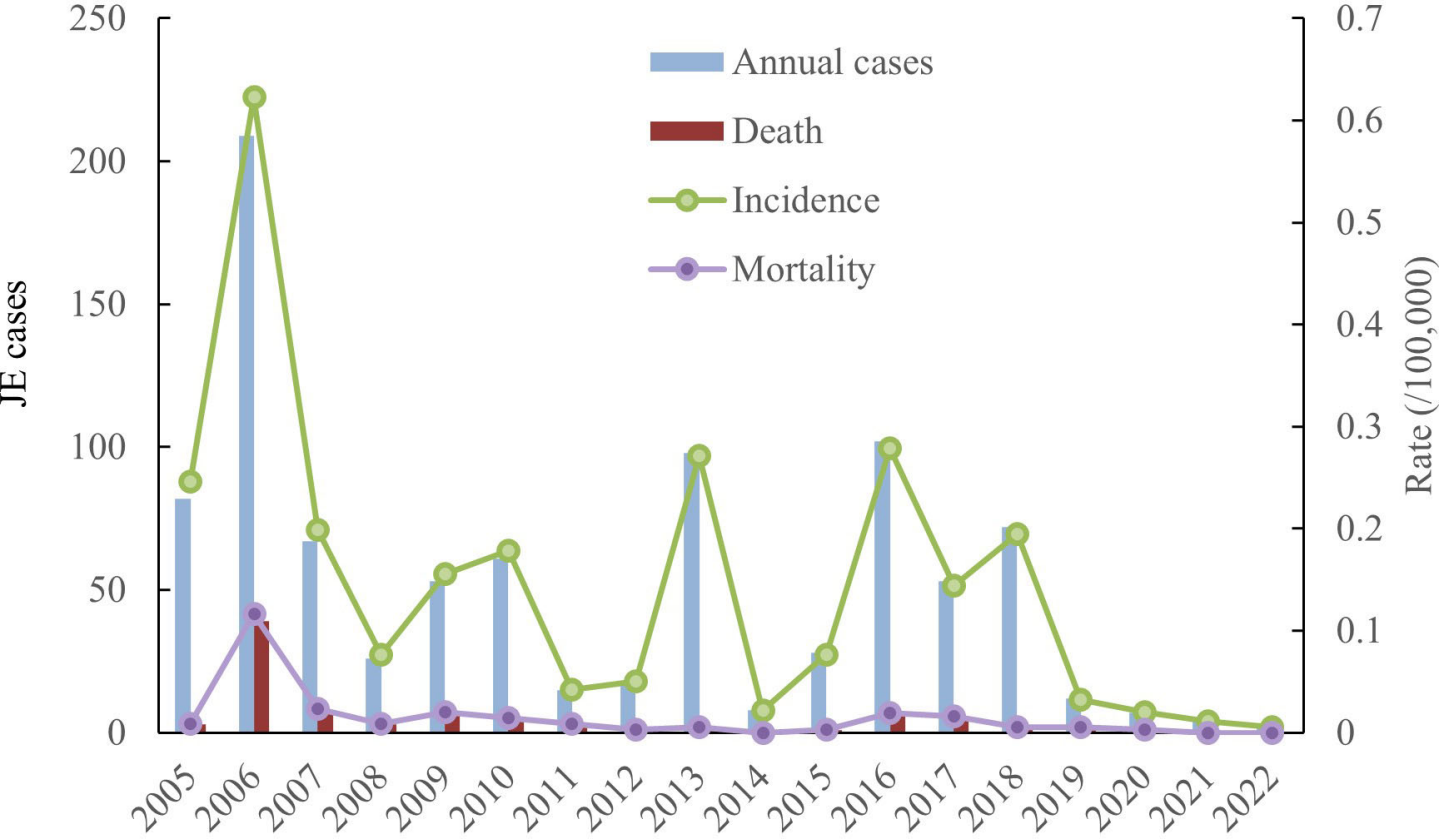
Annual cases and death, incidence and mortality of JE in Shanxi Province,2005–2022.

Incidence and mortality. A total of 917 cases and 90 deaths were documented between 2005 and 2022, with an annual incidence between 0.0057/100,000 and 0.623/100,000, and an annual mortality between 0 and 0.1162/100,000. The average annual incidence and mortality rates were 0.1459/100,000 and 0.0145/100, 000, respectively, with a fatality rate of 9.81%. The incidence of JE in Shanxi Province showed a decreasing trend between 2005 and 2022, with the highest number of JE cases in 2006 (**Figure 1**).

**Figure 2:**
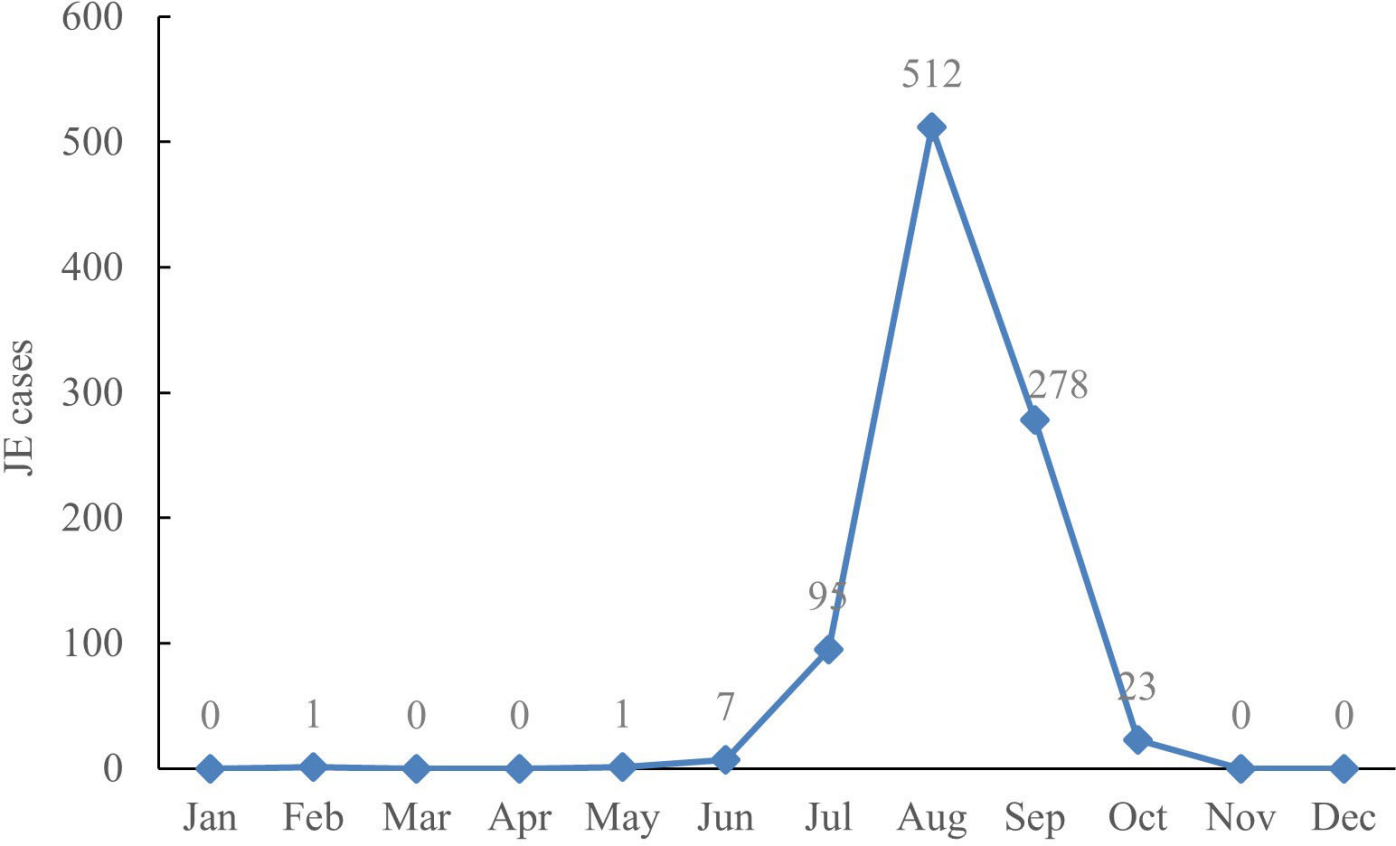
Monthly cumulative JE cases in Shanxi Province, 2005–2022.

Seasonal pattern. The distribution of JE cases revealed clear seasonal patterns, with the highest wave from July to September, with a peak in August. There were 95 cases (10.36%) in July, 512 cases (55.83%) in August, 278 cases (30.32%) in September, and a total of 885 cases (96.51%) from July through September among JE cases between 2005 and 2022 (**Figure 2**).

**Figure 3:**
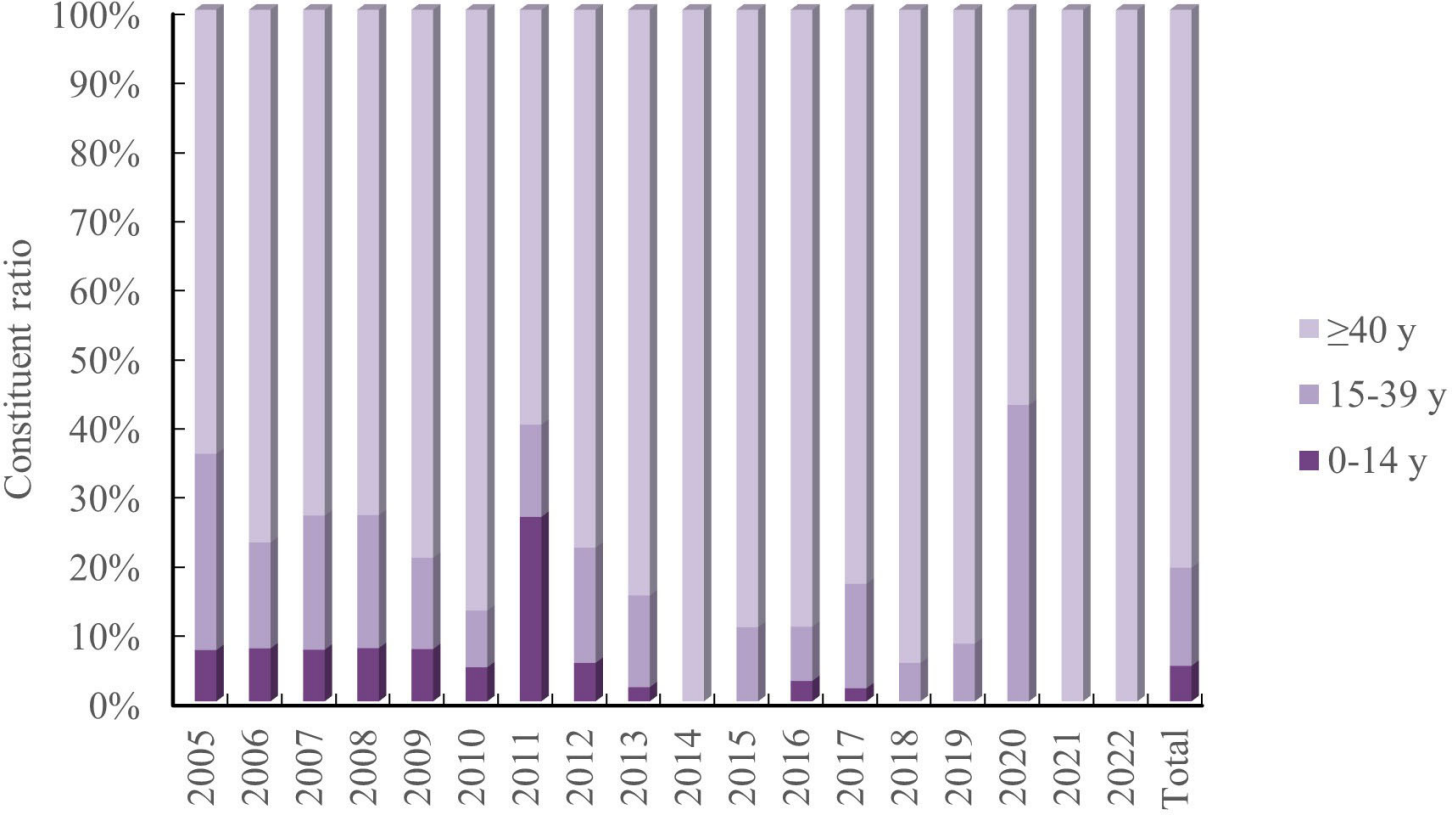
Constituent ratio of JE cases by age group in Shanxi Province, 2005–2022.

Age pattern. There were 47 cases (5.13%), 130 cases (14.18%), and 739 cases (80.59%) that were 0-14 years, 15-39 years, and ≥40 years, respectively, with 1 unknown case. The age group ≥ 40 years had the highest concentration of JE cases, with the age group 0-14 years gradually decreasing. There were no cases in the age group 0-14 years in 2014-2015 and 2018-2022, and all JE cases in 2014 and 2021-2022 were ≥40 years (**Figure 3**).

**Figure 4:**
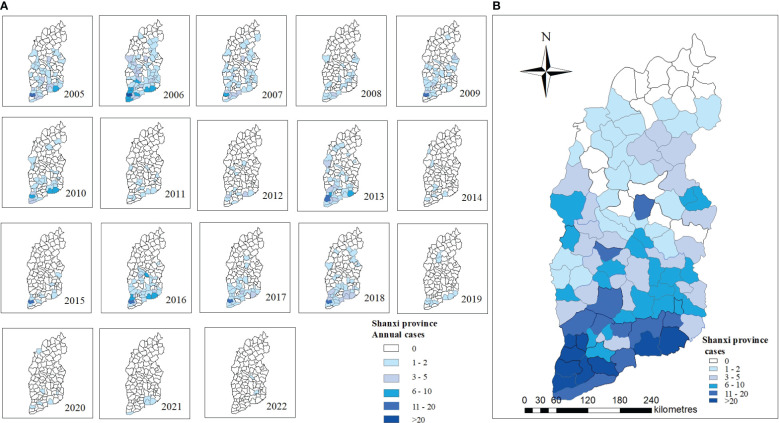
Spatiotemporal distribution of JE in Shanxi Province, 2005–2022. **(A)** Annual JE cases in 2005-2022. **(B)** Total JE cases in 2005-2022.

Geographical distribution. Shanxi Province has 117 counties, with 18 counties reporting no JE for over 10 years. **Figure 4A** depicts the annual distribution of JE cases by county. Liyi, Wanrong, Jincheng urban area (Zezhou), Yangcheng, and Hejin counties had the highest number of reported cases (**Figure 4B**).

### 3.2 Spatial features of JE in Shanxi Province

**Figure 5:**
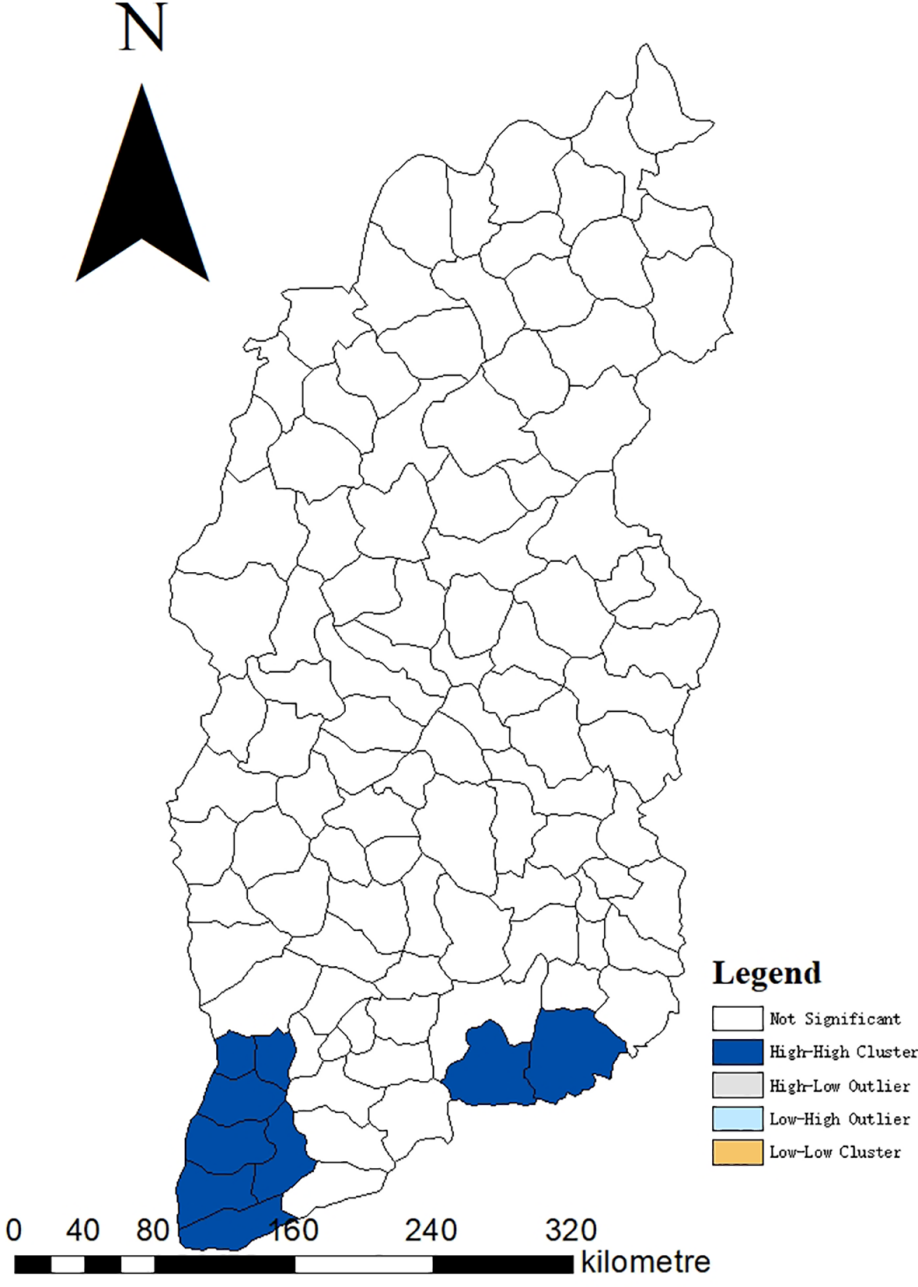
Moran LISA cluster map of JE cumulative cases in Shanxi Province, 2005–2022. LISA, Local Indicators of Spatial Association.

The incidence of JE from 2005 to 2022 had a positive spatial correlation with significant clustering characteristics (Moran’s I=0.39, Z=8.13, P<0.001). LISA revealed significant spatial agglomeration areas, including high-incidence clusters in the south and southeast: Hejin County, Jishan County, Wanrong County, Liyi County, Yongji County, Ruicheng County, Yanhu District, Yangcheng County, Jincheng urban area (Zezhou County) (**Figure 5**).

### 3.3 General information and clinical features

The study comprised 138 patients with JE (**Table 1**),79 (57.25%) of whom were male, the median age was 63 years, and the majority of patients were over 50 years old. The majority of the cases occurred in the summer. There were 101 cases with good outcomes and 37 cases with poor outcomes, including 56 men (55.45%) and 23 men (62.16%), respectively. Hypertension was the most prevalent underlying condition. Compared with the good outcomes group, the poor outcomes group was older (65 vs. 61, p<0.05), but there were no significant differences in gender and underlying diseases (p>0.05).

**Table 1:** General information and clinical features with a comparison between the good and poor outcomes groups.

	Total(n=138)	Good Outcomes (n=101)	Poor outcomes (n=37)	χ2/Z	P
General information					
Male	79 (57.25)	56 (55.45)	23 (62.16)	0.499	0.48
Age	63 (53,70)	61 (52.5,68)	65 (60,75)	-2.332	0.02*
18-29	5	4	1		
30-39	8	6	2		
40-49	11	10	1		
50-59	32	27	5		
60-69	45	32	13		
>70	37	22	15		
Underlying condition					
Hypertension	28 (20.29)	17 (16.83)	11 (29.73)	2.785	0.095
CHD	6 (4.35)	4 (3.96)	2 (5.4)	0.136	0.659
Diabetes	8 (5.80)	5 (4.95)	3 (8.1)	0.494	0.442
Clinical symptoms					
Fever	136 (98.55)	99(98.02)	37 (100)	0.743	1.0
Temperature	39.2 (38.8, 39.6)	39(38.6, 39.4)	39.7 (39.45, 39.9)	-6.694	<0.001*
Headache	87 (63.04)	61(60.4)	26 (70.27)	1.133	0.287
Consciousness disruption	79 (57.25)	44 (43.56)	35 (94.6)	45.853	<0.001*
Somnolence	17 (12.32)	14 (13.86) a	3 (8.1) a	–	–
Stupor	15 (10.87)	10 (9.9) a	5 (13.5) a	–	–
Light coma	26 (18.84)	14 (13.86) a	12 (32.43) b	–	–
Moderate coma	6 (4.35)	2 (1.98) a	4 (10.81) b	–	–
Deep coma	15 (10.87)	4 (3.96) a	11 (29.73) b		
Vomiting	49 (35.51)	35 (34.65)	14 (37.84)	0.12	0.729
Convulsion	38 (27.54)	23 (22.77)	15 (40.54)	4.285	0.038*
Signs					
Meningeal irritation	94 (68.12)	69 (68.32)	25 (67.57)	0.007	0.933
Pathological finding	31 (22.46)	16 (15.84)	15 (40.54)	9.484	0.002*
Muscle tone				13.035	0.001*
rigidity	44 (31.88)	33 (32.7) a	11 (29.73) a	–	–
flaccidity	23 (16.67)	10 (9.9) a	13 (35.14) b	–	–
Muscle weakness	40 (28.99)	26 (25.74)	14 (37.84)	1.925	0.165
Complications					
Pulmonary infection	81 (58.7)	52 (51.49)	29 (78.38)	8.078	0.004*
Respiratory failure	47 (34.06)	26 (25.74)	21 (56.76)	11.598	0.001*

CHD, Coronary heart disease.

a, a: the difference was not statistically significant; a, b: the difference was statistically significant.

* P<0.05

Fever, with a median temperature of 39.2° C, was the most common symptom (98.55%), followed by headache (63.04%) and consciousness disruption (57.25%). Meningeal irritation was the main sign in 94 patients (68.12%); 31 presented with positive pathological findings, 23 had flaccid muscle tone, and 40 had muscle weakness. Temperature, consciousness disruption (coma), convulsions, positive pathological findings, and flaccid muscle tone were associated with poor outcomes. Pulmonary infection and respiratory failure were the most common clinical complications linked to poor outcomes. (**Table 1**)

### 3.4 Laboratory and imaging examination

The CSF was colorless and transparent, with increased pressure. The median CSF white blood cell (WBC) was 91*10^6^/L, with slightly increased protein and reduced chloride. The CSF pressure, protein, and sugar levels in the poor outcomes group were significantly higher than in the good outcomes group (P< 0.05). WBC, neutrophils % (N%) in the poor outcomes group was higher than in the good outcomes group, but albumin (ALB) was lower (**Table 2**).

**Table 2:** Laboratory and imaging examination with a comparison between the good and poor outcomes groups.

	Total (n=138)	Good Outcomes (n=101)	Poor outcomes (n=37)	χ2/t/Z		P	
CSF							
Pressure (mmHg)	190 (165, 231.25)	190 (157.5, 220)	225 (172.5, 290)	-3.019		0.003*	
WBC (10^6^/L)	91 (59.5, 134.25)	89 (56, 133.5)	99 (63, 143)	-5.589		0.556	
Protein (g/L)	0.74 (0.49, 0.91)	0.71 (0.41, 0.88)	0.83 (0.65, 1.23)	-2.802		0.005*	
Sugar (mmol/L)	3.3 (2.99, 3.92)	3.17 (2.93, 3.64)	3.85 (3.18, 4.23)	-3.456		0.001*	
Chloride (mmol/L)	118.88 ± 7.81	119.15 ± 8.35	118.16 ± 6.16	0.756		0.452	
Blood test							
Hb (g/L)	133.72 ± 12.68	135.41 ± 9.37	129.11 ± 18.39	1.99		0.053	
WBC (10^9^/L)	10.9 (8.38, 14.9)	9.6 (7.8, 12.55)	16.6 (13.8, 18.7)	-6.794		<0.001*	
N%	80.95 (73.38, 85.73)	77.5 (71.6, 84.25)	86 (82.95, 88.7)	-5.792		<0.001*	
PLT (10^9^/L)	208.43 ± 40.65	208.63 ± 42.02	207.86 ± 37.2	0.098		0.922	
ALT (U/L)	54.5 (35.75, 80)	50 (35.5, 77)	65 (36.5, 88)	-1.524		0.128	
AST (U/L)	39 (26, 52)	39 (24, 50)	44 (34, 53)	-1.596		0.11	
TBIL (umol/L)	13.55 (9.7, 16.93)	13.5 (9.9, 16.6)	13.6 (7.65, 19.85)	-0.392		0.695	
ALB (g/L)	39.83 ± 5.7	41.16 ± 5.53	36.2 ± 4.47	4.896		<0.001*	
CR (umol/L)	73.49 ± 12.07	74.17 ± 10.77	71.63 ± 15.09	0.938		0.353	
UR (mmol/L)	5.09 (3.87, 6.5)	5.3 (3.9, 6.75)	4.68 (3.74, 5.86)	-1.281		0.2	
Na (mmol/L)	136.33 ± 6.14	136.72 ± 6.41	135.27 ± 5.28	1.233		0.22	
K (mmol/L)	4.04 (3.4, 4.65)	4. 1 (3.31, 4.78)	3.99 (3.42, 4.52)	-0.077		0.939	
Cl (mmol/L)	98.5 (95.3, 104.4)	99.3 (95.6, 105.1)	97.5 (94.85,100.3)	-1.843		0.065	
Brain MRI	79	63	16				
Thalamus	30 (37.97)	22 (34.92)	8 (50)	1.232		0.267	
Hippocampus	16 (20.25)	10 (15.87)	6 (37.5)	2.477		0.115	
Basal ganglia	13 (16.46)	11 (17.46)	2 (12.5)	0.01		0.92	
Mesencephalon	10 (12.66)	7 (11.11)	3 (18.75)	0.16		0.689	
Cerebral hemisphere	8 (10.13)	7 (11.11)	1 (6.25)	0.012		0.911	

CSF: cerebrospinal fluid; WBC: white blood cell; N%: neutrophils%; ALB: albumin.

*: P<0.05.

As illustrated in **Table 2**, 79 patients underwent brain MRI, with 41 (51.9%) indicating inflammatory lesions. The thalamus was the most involved site, followed by the hippocampus and basal ganglia. Some patients involved two or more sites simultaneously. The sites implicated in the two groups differed, but the difference was not statistically significant.

### 3.5 Prognostic factor

**Figure 6:**
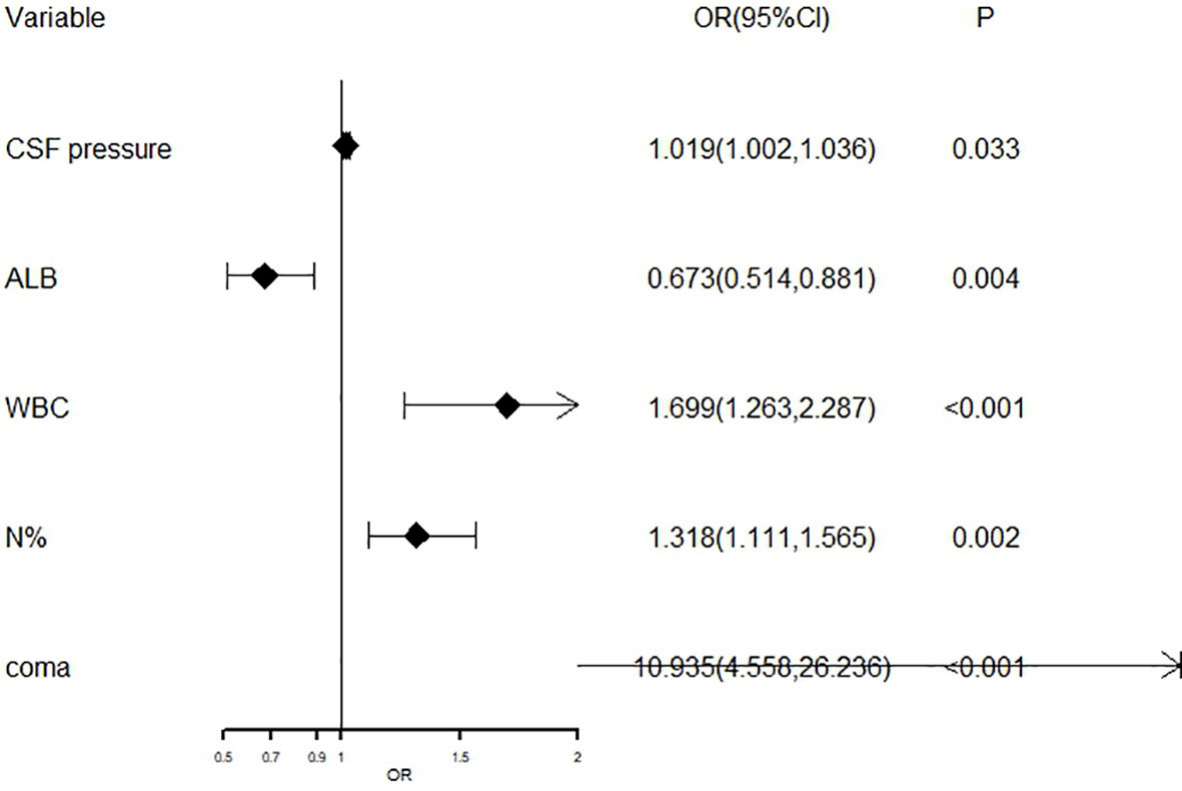
The forest plot for logistic regression analysis in poor outcomes of adult JE CSF, cerebrospinal fluid; ALB, albumin; WBC, white blood cell; N%, neutrophils%.

The aforementioned significant variables in **Tables 1**, **2** were included in the Logistic stepwise regression analysis, with the threshold value α_in_ at 0.05 and α_out_ at 0.10 to screen out five independent predictors of poor outcomes JE patients (**Figure 6**). CSF pressure, WBC, N%, and coma were risk factors for poor outcomes. For 1 unit increase in CSF pressure, WBC, N%, the risk of poor outcomes increased by 1.9%, 69.9%, and 31.8%, respectively, and the risk of poor outcomes increased by 9.935 times in patients with coma. ALB was a protective factor for poor outcomes, and 1 unit increase in ALB reduces the risk by 32.7%.

### 3.6 Evaluation predictive model

**Figure 7:**
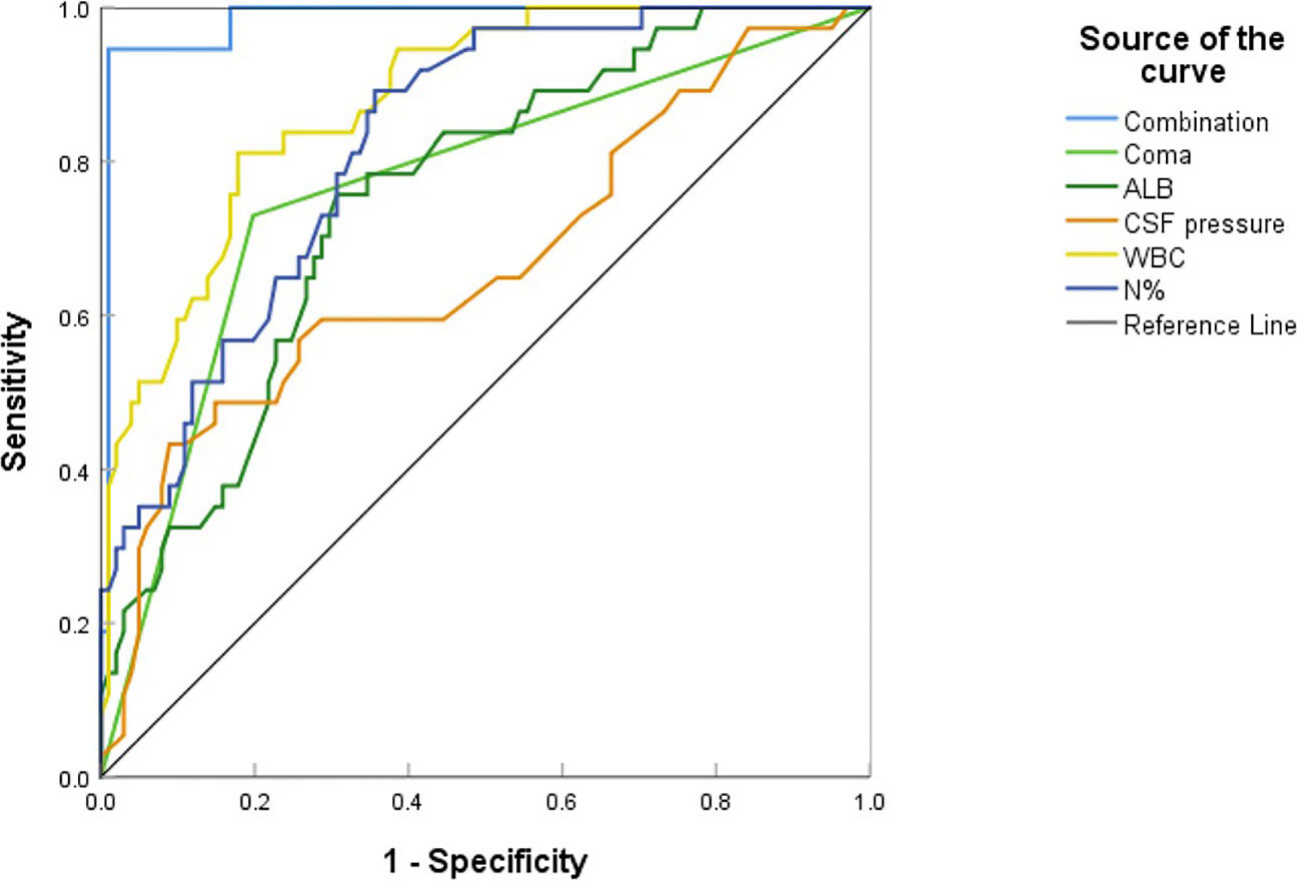
The ROC curve for predictive model of adult JE.

Multivariate analysis revealed that higher CSF pressure, higher WBC, higher N%, deep coma, and lower ALB were independent predictors of poor outcomes, generating a new prediction model: ln(p/1p)=0.019× (CSF pressure)+0.53× (WBC)+0.276× (N%)+2.392× (coma)-0.396× (ALB). The ROC curve was used to assess the prediction efficiency (**Figure 7**), sensitivity, specificity, and Youden index were calculated respectively, and the optimal critical value for prediction of each index was obtained from the maximum Youden index. The AUC of the combined prediction model was 0.983, the sensitivity was 94.6%, and the specificity was 99%, higher than that of CSF pressure (AUC=0.688), WBC (AUC=0.878), N% (AUC=0.822), coma (AUC=0.766), ALB (AUC=0.752) prediction alone (P <0.05) (**Table 3**), so the prediction model had the best prediction efficiency.

**Table 3:** The comparison of prediction efficiency in predictive model.

Variable	Cut Off	AUC (95%CI)	Sensitivity	Specificity	Youden Index	P
coma	–	0.766 (0.671, 0.861)	0.73	0.802	0.532	<0.001
ALB	38.25	0.752 (0.665, 0.838)	0.757	0.693	0.45	<0.001
CSF pressure	247.5	0.668 (0.559, 0.777)	0.432	0.911	0.343	0.003
WBC	13.55	0.878 (0.819, 0.938)	0.811	0.822	0.633	<0.001
N%	80.95	0.822 (0.751, 0.894)	0.892	0.644	0.536	<0.001
Combination	–	0.983 (0.963, 1.000)	0.946	0.99	0.936	<0.001

**Figure 8:**
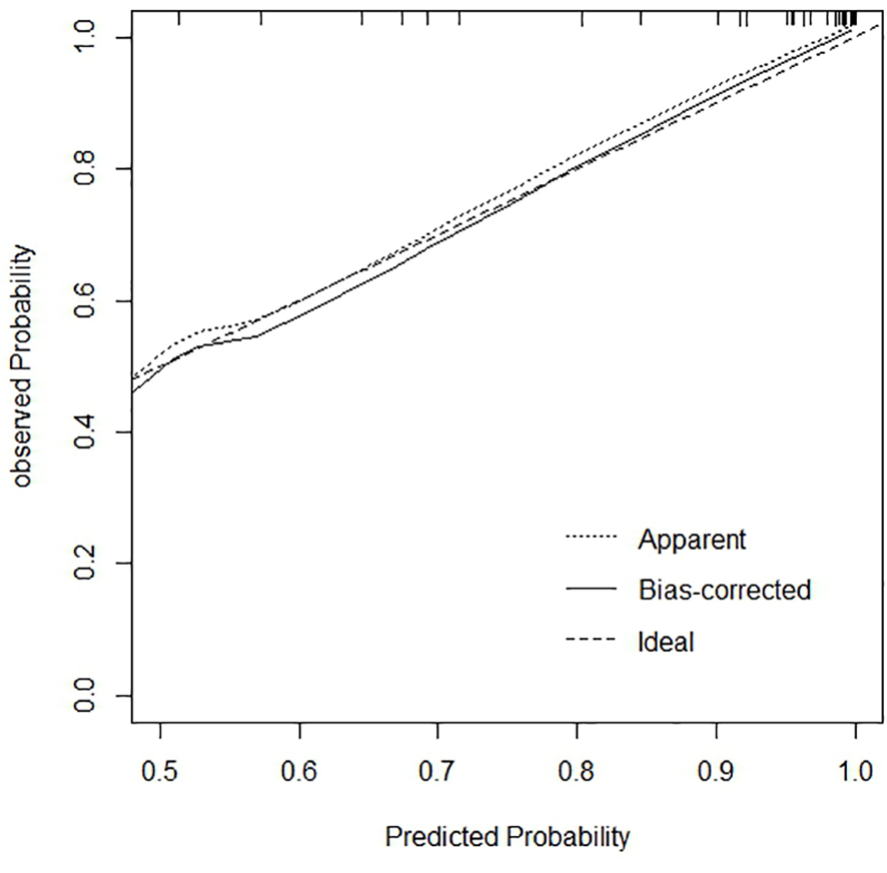
The calibration curve for predictive model of adult JE.

The calibration curve and Hosmer-Lemeshow goodness of fit test were drawn based on the bootstrape1000 repeated sampling verification to evaluate the calibration degree of the model (**Figure 8**). The predicted probability of poor JE outcomes was consistent with the actual probability, and the predicted curve matched the ideal curve. In the Hosmer-Lemeshow test, χ2 = 2.025, P=0.363 (P>0.05), indicating a good fit.

**Figure 9:**
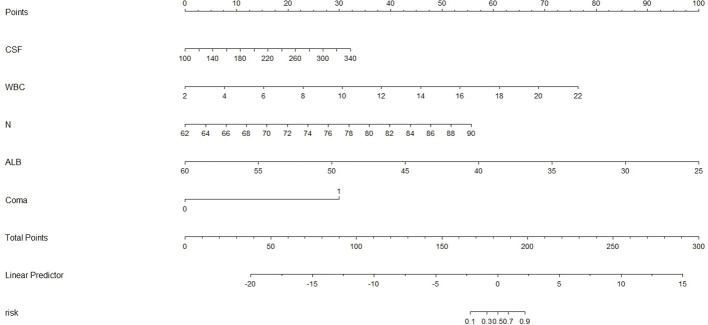
The nomogram for predictive model of adult JE.

We constructed a nomogram for predicting poor JE outcomes using the above five predictors (**Figure 9**). According to the contribution degree of each variable, each value level of the variable corresponded to the upper score, from 0 to 100, then the score of each variable was combined to obtain the total score, and finally revealed the prediction probability of poor outcomes.

## 4 Discussion

This was the first study to examine the epidemiological characteristics of JE distribution in Shanxi Province during the last 18 years, and the prognosis factors of adult JE were investigated using clinical data to develop a prediction model. A total of 917 cases were recorded in Shanxi Province between 2005 and 2022, with an average annual incidence and mortality of 0.1459/100,000, 0.0145/100,000, and a fatality rate of 9.81%. These cases primarily occurred in August and September, with 739 cases (80.59%) involving people over the age of 40. Li and colleagues found that the proportions of JE for the above 40-year-old group in the six provinces north of the Yangtze River (Henan, Hebei, Shandong, Shanxi, Shaanxi, and Gansu) between 2004 and 2014 were higher than the national average (Li et al., 2016). Shanxi province accounted for over 50% of all JE cases in the over-40-year-old group, higher than the national average.

Shanxi Province has 18 counties with no recorded JE cases between 2005 and 2022, the majority of which were in northern Shanxi Province. JE prevention has been intensively pursued for many years; however, JE cases were found in most parts of Shanxi Province. The global spatial autocorrelation revealed a positive spatial correlation with significant clustering characteristics, indicating that disease burden was unevenly distributed across counties in Shanxi Province. LISA revealed the presence of significant spatial agglomeration areas, with high-incidence clusters in the south and southeast, primarily counties under the jurisdiction of Yuncheng City. Yuncheng City, located along the Yellow River and in the southernmost part of Shanxi Province, with high temperature and humidity, has a high prevalence of JE. Studies have demonstrated that temperature and relative humidity directly influence the distribution and population dynamics of mosquitoes. Temperature affects mosquito development and viral replication in an amplifying host and vector, and appropriate relative humidity allows mosquitoes to survive longer and spread further, increasing the risk of pathogen transmission (Song et al., 2020; Tu et al., 2021). A study in Liyi County, Yuncheng City, found that cotton field water by periodic flooding generates abundant favorable breeding sites for JE vector mosquitoes, and cotton field irrigation management was beneficial for JE control in this region (Liu et al., 2022). Furthermore, there was a tight association between pig number, vector infection rate, and the number of human JE cases (Borah et al., 2013). Pigsties near human dwellings serve as an amplifying host for JEV, resulting in JEV proliferation in the surrounding areas and an increased risk of JEV infection in humans. The high mortality caused by the JE outbreak in Yuncheng City in 2006 drew attention. Therefore, the pigsties and environment should be strengthened to minimize the incidence of adult JE in the high-incidence area.

Recently, there has been a great amount of support for vaccinating adults against JE. Supporters believe that it is urgent to carry out vaccination for adults based on the number of adult patients and the age of onset(Mao and Zhou, 2020). Their claim is unrealistic and unsustainable for all adults to be vaccinated, at least in Shanxi Province, because the hot spots for the JE epidemic were in the south and southeast. Therefore, adults at high risk of JEV exposure such as those working in rural agricultural areas or pig rearing businesses are recommended to receive the JE vaccine. Although the incidence and mortality of JE have shown a downtrend in Shanxi Province, China, the risk of an epidemic or an outbreak is still high. In recent years, we established a strong system for epidemic reporting, and the system has been running smoothly. However, most cases of JE are asymptomatic and go unreported, which makes the spatial distribution of the virus difficult to estimate. In a study that was reported in the south of Shanxi Province (Ren et al., 2017), the minimum infection rate of JEV in mosquito specimens collected from the courtyards of farmers’ households with pigsties was 7.39/1 000. In addition, agricultural changes; climate changes; JEV infection without encephalitis; changes in trip duration for JE case can also cause an outbreak or epidemic. This study suggested that the risk of JE epidemic occurring is still high and that prevention efforts in Shanxi Province should not be neglected.

Because of the rising proportion and poor prognosis of adult JE patients in Shanxi Province, this study analyzed clinical cases to identify prognosis factors of adult JE patients. There were somewhat more men than females in these clinical cases, but gender did not affect JE prognosis (Lo et al., 2019; Chen et al., 2022). These older patients had poor prognoses, possibly due to organ function decline, decreased immunity, and poor overall health (Li et al., 2022). The predominant clinical symptoms were fever, headache, vomiting, and consciousness disruption. Fever is the most common symptom (Gu et al., 2022), and patients with poor prognosis experienced elevated body temperatures. The consciousness disruption of is characterized by a partial or complete loss of conscious activity, which is related to the severity of brain damage. Coma is the complete loss of consciousness. Univariate analysis demonstrated that coma and convulsion are risk factors for poor JE prognosis, and recurrent convulsion and coma are indicators of nervous system involvement. Previous research evidence linked coma to poor prognosis of severe JE (Su et al., 2021; Chen et al., 2022). Dystonia is primarily caused by damage to the structure or network of the extrapyramidal system of the nervous system. Intracranial lesions in JE patients were located mainly in the thalamus, basal ganglia, and other parts, and muscle tone flaccidity is associated with poor prognosis, which is consistent with the findings of Lo et al. (2019). Because the above indicators of nervous system involvement imply that some patients have nontypical initial symptoms, we should pay close attention to nervous system examination to detect abnormal signs, identify early cases, and improve clinical prognosis.

Pulmonary infection and respiratory failure are the most common complications of JE, and univariate analysis revealed their potential link with poor prognosis. Most patients with consciousness disruption, long-term bed rest, poor cough ability, and low immunity increase the probability of lung infection. Respiratory failure is primarily caused by brain parenchymal lesions involving the respiratory center, necessitating mechanical ventilation, with ventilator-related pneumonia being the most prevalent complication. Patients with lung infection require additional supportive care and have poor prognoses (Ma and Jiang, 2013). Xiong et al. (2019) revealed that older JE patients had a higher incidence of acute secondary complications, including respiratory failure, hypoalbuminemia, and a poor prognosis. Therefore, these complications require early diagnosis, treatment, and prevention by ensuring smooth breathing and taking appropriate measures such as tracheal intubation and tracheotomy, combined with respiratory stimulants and glucocorticoids, as needed.

Nearly 50% of JE patients had increased CSF pressure and a poor prognosis. JEV infection can induce endothelial cells and astrocytes to produce mediators that regulate JEV production while destroying the integrity of the blood-brain barrier (Patabendige et al., 2018), increasing the CSF protein. A previous study discovered that CSF protein levels were linked to neurological symptoms, suggesting that abnormal intrathecal protein synthesis results in poor prognosis (Sarkar et al., 2015). Furthermore, CSF sugar levels in patients with JE were either normal or slightly elevated. Zheng (2020) demonstrated that CSF sugar was an independent factor of poor prognosis, indicating disease severity. JEV entrance into the body can induce oxidative stress reactions (such as reactive oxygen species, superoxide anion, and NO production) in neutrophils and glial cells, causing inflammation, immune activation, autophagy, and other cellular reactions (Sharma et al., 2018). Activation of the innate immune response inhibits viral spread and promotes the release of inflammatory mediators, and lectin expression on macrophages and neutrophils potentially exacerbates JEV-mediated neuroinflammation (Chen et al., 2012). An increase in WBC and N% was associated with poor prognosis. Su et al. demonstrated that a higher percentage of neutrophils and a lower percentage of lymphocytes on admission may be associated with poor prognosis (Su et al., 2021). Other research has linked a high WBC count to poor neurological prognosis (Kafle et al., 2017). Low ALB may be attributed to long-term bed rest, insufficient protein intake, malnutrition, or consumption status, resulting in decreased immunity; thus, a normal protein level is a protective factor for prognosis, which warrants adequate energy provision through nutritional support therapy.

Multivariate logistic regression analysis revealed that higher CSF pressure, higher WBC, higher N%, deep coma, and lower ALB were independent factors for poor JE outcomes. The prediction model had an AUC of 0.983, with 94.6% sensitivity and 99% specificity. Generally, the AUC between 0.5-0.7 implies poor accuracy, 0.7-0.9 moderate accuracy, and 0.9 high accuracy. In this view, the prediction model used in this investigation was highly accurate. Analysis of prognostic factors in adult JE in Taiwan revealed that CSF protein and dystonia were independent factors of poor prognosis (Lo et al., 2019). Another investigation of predictors of poor outcomes among JE survivors demonstrated that subjects who did not receive JE vaccination, were malnourished, had GCS ≤8 at admission, and required endotracheal intubation, had poorer outcomes with 77.8% sensitivity and 94.6% specificity (Srivastava et al., 2022). Sunwoo et al. (2017) demonstrated that midbrain injury and rapid disease deterioration were linked to severe neurological sequelae.

The calibration curve reflects the predicted probability of the prediction model consistency with the actual probability. Our prediction curve was consistent with the ideal curve, indicating that the prediction probability of the model concurred with reality, with high accuracy in predicting the poor prognosis of JE. Evaluating the goodness of fit of the logistic regression model is the key to the accuracy of estimated probabilities (Nattino et al., 2020). The Hosmer-Lemeshow test (P>0.05) revealed that the model was fitted and highly accurate. A visual nomogram was generated based on the five factors of JE poor prognosis, which can compute the total score according to the numerical value of each variable and then reveal the prediction probability of poor outcomes, providing clinicians with an early assessment of the risk of poor prognosis and make targeted prevention. Patients with increased CSF pressure, for example, should actively lower cranial pressure to prevent the occurrence of brain death and brain hernia. Patients with increased WBC and N% should consider early antibiotic usage to prevent and manage infection. These patients should also receive adequate nutritional support therapy. Early supportive treatment can limit the progression of JE and improve prognosis significantly. At the same time of basic treatment, strict control of body temperature, early invasive ventilator support therapy, and early consciousness disorder rehabilitation training can achieve significant curative effect (Gao et al., 2021). The comprehensive ICU treatment (hormones combined with anti-inflammatory, antiviral, and mild hypothermic cerebral protection therapies) can improve the survival rate(Gu et al., 2022). However, the effect of steroid use on outcome improvement remained inconsistent among studies. Lo et al. showed that no significant differences were observed between the good outcomes group and poor outcomes group in terms of steroid or antiviral treatments(Lo et al., 2019).

There are some limitations to this study. The number of JE patients in Shanxi Province has gradually declined in recent years due to economic growth, environmental improvement, mosquito control efforts, and increased public awareness of JE vaccination. Therefore, it is difficult to collect data on the long-term prognosis of patients after discharge due to the extended time range to collect enough cases retrospectively; thus, we used the GCS score at discharge to evaluate the prognosis. GCS score at admission has been linked to mortality, while GCS score at discharge is statistically significant for long-term prognosis. (Zhang et al., 2023). Second, due to the small sample size of the included studies and potential statistical errors in the model prediction procedure, the multivariate analysis of poor prognosis should be validated in the future using multi-sample and multi-center analyses. In addition, our study included limited influencing factors and some confounding factors that may have been overlooked. The different indicators and timely diagnosis and treatment influence patient prognosis in clinical practice.

## 5 Conclusion

Our study showed the epidemic dynamics and hotspots of JE in Shanxi Province. July to September was the primary epidemic season of JE and that JE cases were mainly in individuals over the age of 40. The age distribution of JE is steadily becoming more adult, with more severe complications and mortality and a higher disease burden, making adult JE a public health concern. Higher cerebrospinal fluid pressure, higher white blood cell counts, higher neutrophil percentage, deep coma and lower albumin were independent factors for poor outcomes of adult JE. The developed risk prediction model holds great promise in early prognosis assessment of patients, providing a basis for clinical decision-making and early clinical intervention.

## Data availability statement

The original contributions presented in the study are included in the article/supplementary material. Further inquiries can be directed to the corresponding author.

## Ethics statement

Ethical approval was not required for the study involving humans in accordance with the local legislation and institutional requirements. Written informed consent to participate in this study was not required from the participants or the participants’ legal guardians/next of kin in accordance with the national legislation and the institutional requirements.

## Author contributions

PZ : Writing – original draft. ZW: Writing – original draft. YL: Writing – review & editing. QW: Writing – review & editing.
